# Epidemiology of lichen sclerosus in women: Findings from the Danish skin cohort

**DOI:** 10.1016/j.jdin.2026.04.019

**Published:** 2026-05-08

**Authors:** Jeanette Halskou Haugaard, Lea Nymand, Yuki MF. Andersen, Mia-Louise Nielsen, Jacob P. Thyssen, Alexander Egeberg

**Affiliations:** a Department of Dermatology and Venerology, Bispebjerg Hospital, Copenhagen, Denmark; b Department of Clinical Medicine, Faculty of Health and Medical Sciences, University of Copenhagen, Copenhagen, Denmark

**Keywords:** epidemiology, lichen sclerosus, underdiagnosed

*To the Editor:* Lichen sclerosus (LS) is a chronic inflammatory dermatosis that predominantly affects the anogenital region and most commonly affects women. LS can cause structural changes and may lead to carcinoma. Common symptoms include pruritus and pain.^1^ This study aimed to describe the diagnostic journey and clinical characteristics of LS in women in a nationwide setting.

Data are from the Danish skin cohort, a prospective cohort established to investigate the natural history of skin diseases in adults. The cohort has been described in detail elsewhere.^2^ This study included female patients diagnosed with LS by a dermatologist or gynecologist between 2000 and 2022. Only patients with LS disease activity within the past 12 months were included. Patients were systematically interviewed regarding their LS. Bimodality was supported by Gaussian mixture modeling, selected using Bayesian Information Criterion (ΔBIC >10).

The study included 2040 female patients with active LS and 2040 age-matched and sex-matched controls. Baseline characteristics of the patients and controls are presented in Table I. The median age at LS symptom onset was 50.0 years (interquartile range, 35.0-57.0) with a bimodal distribution showing peaks at 24.2 (standard deviation: 10.3) and 53.2 years (standard deviation: 9.2). Most patients were diagnosed by a gynecologist (80.2%), followed by a dermatologist (22.3%), and other physicians, including general practitioners (14.8%). The mean time from symptom onset to LS diagnosis was 4.8 years, shortest for patients diagnosed by a dermatologist or other physician (4.0 years and 3.9 years), longest for urologists (8.1 years). We observed a significant association between age of symptom onset and diagnostic delay of LS (*P*-trend < .0001).

**Table I:** Baseline characteristics of female patients with lichen sclerosus and controls

Characteristics	Patients with LS (*n* = 2040)	Controls (*n* = 2040)
Age in y, median (IQR)	65.0 (56.0-72.0)	65.0 (56.0-72.0)
BMI, mean (SD)	26.5 (5.5)	27.0 (11.9)
Physical activity, *n* (%)		
Sedentary	350 (17.2)	364 (17.8)
Moderate	1238 (60.7)	1144 (56.1)
Vigorous	422 (20.7)	426 (20.9)
Athletic	6 (0.3)	10 (0.5)
Unknown/missing	24 (1.2)	29 (1.4)
Smoking status, *n* (%)		
Current daily smoker	101 (5.0)	201 (9.9)
Current occasional smoker	34 (1.7)	47 (2.3)
Former smoker	845 (41.4)	779 (38.2)
Never smoker	1052 (51.6)	943 (46.2)
Unknown/missing	8 (0.4)	10 (0.5)
Alcohol consumption (within past 12 mo), units/wk, mean (SD)	3.1 (12.1)	4.2 (6.5)
Sleep disturbance (NRS), mean (SD)	3.4 (2.7)	3.0 (2.6)
Itch intensity on NRS, mean (SD)	2.9 (2.7)	1.7 (2.1)

*BMI*, Body mass index; *IQR*, interquartile range; *LS*, lichen sclerosus; *NRS*, numeric rating scale; *SD*, standard deviation.

Fig 1 illustrates the complex patient journey from symptom onset to diagnosis. A high proportion of patients (*n* = 297, 14.6%) reported waiting over a decade before being diagnosed.

**Fig 1:**
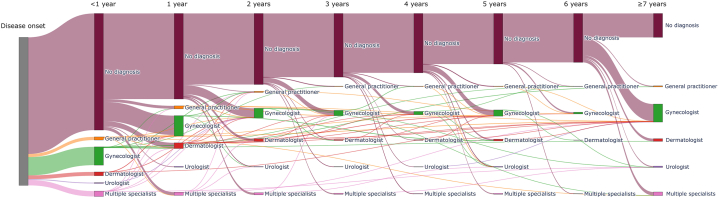
Sankey diagram illustrating the complex health care pathway experienced by patients with lichen sclerosus, beginning with the onset of symptoms and ending with the final diagnosis, often involving multiple consultations with different physicians. As shown, many patients still remain undiagnosed after more than 7 years of symptoms.

We observed a bimodal age distribution of LS symptom onset, with peaks in the 20s and 50s. Previous studies reported a first peak in childhood and a second peak in the 50s.^3^^,^^4^ However, our results are not directly comparable, as our study design cannot assess whether a childhood peak exists, as only adults were included and symptom onset relied on retrospective recall.

Diagnostic delay is observed and likely reflects both patient factors, such as embarrassment in seeking care and health care factors, with many patients consulting their general practitioner but remaining undiagnosed or misdiagnosed.^5^ We observed that lower age at symptom onset was associated with longer diagnostic delay of LS; while our study design does not allow causal inference, this may reflect misattribution of symptoms in younger individuals and fewer opportunities for incidental detection through routine health care.

This study has limitations, including over-representation of more severe LS cases, due to the hospital-based design of the cohort, and the risk of recall bias. Histopathological data were unavailable; however, LS is primarily diagnosed clinically, and biopsy is reserved for selected cases. Strengths include universal access to health care in Denmark, limiting socioeconomic selection bias, and a large sample size.

The clinical implications of diagnostic delay are significant, as optimal management relies on timely diagnosis and prompt initiation of topical corticosteroids. Increased physician awareness of this delay and the complex health care pathway may facilitate earlier treatment, helping to prevent irreversible complications.

## Conflicts of interest

Prof. Thyssen has been an advisor for AbbVie, Almirall, Arena Pharmaceuticals, OM Pharma, Aslan Pharmaceuticals, Union Therapeutics, Eli Lilly & Co, LEO Pharma, Pfizer, Regeneron, and Sanofi-Genzyme; a speaker for AbbVie, Almirall, Eli Lilly & Co, LEO Pharma, Pfizer, Regeneron, and Sanofi-Genzyme; and received research grants from Pfizer, Regeneron, and Sanofi-Genzyme. He is currently an employee at LEO Pharma. Prof. Egeberg has received research funding from Almirall, Pfizer, Eli Lilly, Novartis, Bristol-Myers Squibb, AbbVie, Janssen Pharmaceuticals, Boehringer Ingelheim, the Danish National Psoriasis Foundation, the Simon Spies Foundation, and the Kgl Hofbundtmager Aage Bang Foundation and honoraria as consultant and/or speaker from Amgen, AbbVie, Almirall, Leo Pharma, Zuellig Pharma Ltd, Galápagos NV, Sun Pharmaceuticals, Samsung Bioepis Co, Ltd, Pfizer, Eli Lilly and Company, Novartis, Union Therapeutics, Galderma, Dermavant, UCB, Mylan, Bristol-Myers Squibb, McNeil Consumer Healthcare, Horizon Therapeutics, Boehringer Ingelheim, and Janssen Pharmaceuticals. He is currently an employee at LEO Pharma. Dr Haugaard, Mrs Nymand, Dr Andersen, and Mrs Nielsen have no conflicts of interest to declare.

## References

[1] De Luca D.A., Papara C., Vorobyev A. (2023). Lichen sclerosus: the 2023 update. Front Med (Lausanne).

[2] Egeberg A., Andersen Y.M.F., Thyssen J.P. (2019). Prevalence and characteristics of psoriasis in Denmark: findings from the Danish skin cohort. BMJ Open.

[3] Singh N., Ghatage P. (2020). Etiology, clinical features, and diagnosis of vulvar lichen sclerosus: a scoping review. Obstet Gynecol Int.

[4] Powell J., Wojnarowska F. (2001). Childhood vulvar lichen sclerosus: an increasingly common problem. J Am Acad Dermatol.

[5] Clarke L., Leatherland R., Simpson R.C. (2026). A systematic review of the barriers to diagnosis of vulval lichen sclerosus in primary care. Clin Exp Dermatol.

